# Reduced Fractalkine Levels Lead to Striatal Synaptic Plasticity Deficits in Huntington’s Disease

**DOI:** 10.3389/fncel.2020.00163

**Published:** 2020-06-18

**Authors:** Anya Kim, Esther García-García, Marco Straccia, Andrea Comella-Bolla, Andrés Miguez, Mercè Masana, Jordi Alberch, Josep M. Canals, Manuel J. Rodríguez

**Affiliations:** ^1^Department of Biomedical Sciences, Faculty of Medicine and Health Sciences, University of Barcelona, Barcelona, Spain; ^2^Institute of Neurosciences, University of Barcelona, Barcelona, Spain; ^3^August Pi i Sunyer Biomedical Research Institute, Barcelona, Spain; ^4^Network Center for Biomedical Research in Neurodegenerative Diseases, Barcelona, Spain; ^5^Laboratory of Stem Cells and Regenerative Medicine, Department of Biomedical Sciences, Faculty of Medicine and Health Science, University of Barcelona, Barcelona, Spain; ^6^Production and Validation Center of Advanced Therapies (Creatio), Faculty of Medicine and Health Science, University of Barcelona, Barcelona, Spain

**Keywords:** huntingtin, CX3CL1, microglia, corticostriatal pathway, synaptic pruning, human iPSC

## Abstract

Huntington’s disease (HD) is an inherited neurodegenerative disorder in which the striatum is the most affected brain region. Although a chronic inflammatory microglial reaction that amplifies disease progression has been described in HD patients, some murine models develop symptoms without inflammatory microglial activation. Thus, dysfunction of non-inflammatory microglial activity could also contribute to the early HD pathological process. Here, we show the involvement of microglia and particularly fractalkine signaling in the striatal synaptic dysfunction of R6/1 mice. We found reduced fractalkine gene expression and protein concentration in R6/1 striata from 8 to 20 weeks of age. Consistently, we also observed a down-regulation of fractalkine levels in the putamen of HD patients and in HD patient hiPSC-derived neurons. Automated cell morphology analysis showed a non-inflammatory ramified microglia in the striatum of R6/1 mice. However, we found increased PSD-95-positive puncta inside microglia, indicative of synaptic pruning, before HD motor symptoms start to manifest. Indeed, microglia appeared to be essential for striatal synaptic function, as the inhibition of microglial activity with minocycline impaired the induction of corticostriatal long-term depression (LTD) in wild-type mice. Notably, fractalkine administration restored impaired corticostriatal LTD in R6/1 mice. Our results unveil a role for fractalkine-dependent neuron-microglia interactions in the early striatal synaptic dysfunction characteristic of HD.

## Introduction

Huntington’s disease (HD) is an inherited neurodegenerative disorder characterized by motor, cognitive, and psychiatric disturbances. It is caused by a CAG repeat expansion in the huntingtin gene, leading to abnormal toxic aggregates of mutant huntingtin (mHtt). Neuropathology of HD is prominent in the striatum, the main primary input of basal ganglia (Morigaki and Goto, 2017) and the cerebral cortex, which extensively projects glutamatergic afferents to medium spiny neurons (MSNs) in the striatum. HD disrupts the mechanisms by which cortical and MSNs communicate before the onset of symptoms (Unschuld et al., 2012; Burgold et al., 2019), making corticostriatal pathway dysfunction a keystone in the pathophysiology of the disease (Miller et al., 2011; Wilkes et al., 2019). Some data from HD mouse models suggest that deficits in synaptic plasticity and neuronal processing are the cellular basis for this specific neurodegeneration. For example, loss of corticostriatal synapses (Deng et al., 2013), altered striatal long-term depression (LTD) (Cummings et al., 2007; Creus-Muncunill et al., 2019; Ghiglieri et al., 2019) and reduction of striatal excitatory postsynaptic currents (Cepeda et al., 2003) have been described in R6/1 mice, a transgenic model of HD. This progressive corticostriatal communication impairment markedly alters basal ganglia circuitry activity, ultimately leading to motor disturbances (Hong et al., 2012; Puigdellívol et al., 2015; Veldman and Yang, 2018).

Microglia, the resident immune cells of the CNS, are activated in the brain of HD patients years before clinical manifestations appear (Tai et al., 2007). Evidence suggests that early microglial activation is triggered by mHtt-mediated excitotoxicity and is one of the mechanisms mediating HD pathogenesis (Tai et al., 2007; Yang et al., 2017). However, the nature of such activation remains elusive and whether it presents beneficial or detrimental consequences in HD is still a matter of study. On the one hand, microglia in the striatum of patients and some HD models proliferate and present a classical inflammatory morphological phenotype (Sapp et al., 2001). These cells release neurotoxic pro-inflammatory cytokines, which results in microglial over-activation that finally aggravate HD progression (Crotti et al., 2014). On the other hand, activated microglia also produce anti-inflammatory cytokines and growth factors with trophic and neuroprotective functions (Yang et al., 2017). In HD, these contradictory functions of microglia can be found simultaneously. Thus, transforming growth factor beta (TGF-β) and vascular endothelial growth factor (VEGF), two characteristic biomarkers of alternative non-inflammatory microglial activation (Liu et al., 2013; Yang et al., 2017), have been reported to co-exist with pro-inflammatory cytokines in HD plasma and post-mortem brain tissue (Battaglia et al., 2011; Di Pardo et al., 2013; Chang et al., 2015). Additionally, HD microglial cells express high levels of mHtt (Ferrante et al., 1997) which induces cell-autonomous microglial pathogenic mechanisms (Crotti et al., 2014; Creus-Muncunill and Ehrlich, 2019; Siew et al., 2019). For example, mHtt impairs microglial motility in response to ATP in HD mice (Kwan et al., 2012) an impairment also observed in CX3CR1-KO mice (Pagani et al., 2015). This cellular dysfunction introduces a new element of complexity to the unclear role of microglia in HD.

In the healthy brain, microglia are continuously and actively scanning the brain environment, moving their processes and sensing neuronal activity without perturbing neuronal networks. Surveillant microglia phagocytose inactive synapses (Wake et al., 2009; Schafer et al., 2012) via expression of traditional immune complement molecules (Wake et al., 2013; Hong et al., 2016), and promote synapse and/or spine formation during development and after injury (Parkhurst et al., 2013; Miyamoto et al., 2016) and sensory experience (Tremblay et al., 2010) contributing to sculpt neuronal circuits.

In physiological conditions neurons specifically release fractalkine (FKN) to maintain the surveillant state of microglia (Ransohoff and Perry, 2009). FKN, also known as CX3CL1, is expressed as a membrane-bound glycoprotein that is proteolytically cleaved to release a soluble chemokine (Harrison et al., 1998). FKN modulates microglial motility, migration and activation by interaction with CX3CR1, its only specific receptor that in the brain parenchyma is restricted to microglia (Cardona et al., 2006; Chiu et al., 2013). The FKN-CX3CR1 axis not only contributes to maintaining the surveillant state of microglia but also has a role in modulating synaptic plasticity and neuronal survival (Rogers et al., 2011; Lauro et al., 2015; Luo et al., 2019). In the hippocampus, FKN-CX3CR1 interactions prevent glutamate-mediated excitotoxicity and apoptosis (Deiva et al., 2004) modulate glutamatergic synapses (Ragozzino et al., 2006; Paxinos and Franklin, 2012), and promote adenosine release by microglia (Lauro et al., 2010). Under pathological conditions FKN has conflicting effects on microglia, being neuroprotective and anti-inflammatory in some contexts, while pro-inflammatory and neurotoxic in others (Lauro et al., 2015). Therefore, abnormalities in the FKN-CX3CR1 axis may lead to dysfunction of non-inflammatory microglial activity and contribute to the pathological processes described in HD.

We hypothesized that changes in FKN-CX3CR1 interactions contribute to the striatal synaptic plasticity dysfunction characteristic of HD from early stages of the disease. To validate our hypothesis we used post-mortem striatal samples of HD patients, human induced pluripotent stem cells (hiPSC) derived striatal neurons, and R6/1 mice. This HD mouse model is extensively characterized, exhibiting an onset of motor symptoms from 14 to 15 weeks of age and a progressively severe disease phenotype (Mangiarini et al., 1996; Puigdellívol et al., 2015). Our results unveil a role for FKN-dependent neuron-microglia interactions in the early striatal synaptic dysfunction characteristic of HD.

## Materials and Methods

### HD Mouse Model

Male and female R6/1 transgenic mice expressing the human exon-1 of mHtt containing 115 CAG repeats and their corresponding wild-type (WT) littermates were obtained from Jackson Laboratory (Bar Harbor, ME, United States) and maintained in a B6CBA background. Polymerase chain reaction (PCR) from tail biopsy samples was used to determine genotypes. Animals were housed together in groups of mixed genotypes and kept under a 12:12 h light/dark cycle in a room at 19–22°C and 40–60% humidity, with free access to food and water. Mouse identification and data analysis were recorded by microchip mouse number. All animal procedures were approved by the animal experimentation Ethics Committee of the University of Barcelona in accordance with the regulations established by the Catalan government, the Spanish regulation (RD 53/2013) and European guidelines (2010/63/UE for the care and use of laboratory animals.

### Human Post-mortem Nervous Tissue

Human post-mortem tissue samples of putamen were used to assess FKN expression and concentration. These samples were obtained from the Neurological Tissue Bank of the Biobanc-Hospital Clínic-Institut d’Investigacions Biomèdiques August Pi i Sunyer (IDIBAPS, Barcelona, Spain^1^), following the guidelines and approval of the Ethics Committee of the University of Barcelona and the European ethical guidelines. Samples were collected at autopsy from individuals who had suffered a clinical history of HD (*n* = 8, age: 62.5 ± 7.2 years; postmortem intervals of 4–18 h), and from non-HD controls (*n* = 12, age: 54.5 ± 6.5 years; postmortem intervals of 4–17 h). Samples were fresh-frozen and stored at −80°C for quantitative real-time PCR (qRT-PCR) and western blot analysis.

### Human iPSC Culture, Differentiation and Immunostaining

Two human iPSC lines were employed in this study. Firstly, the CS83iCTR-33nXX (CTR33) line was used, which was derived from an unaffected sibling of a HD patient with genotyped CAG repeat length of 33 in the HTT gene. This line was reprogrammed using a non-integrating strategy and was developed as a “control” line for a related study on HD hiPSC characterization (Telezhkin et al., 2018). Secondly, the CS21iHD60n5 (HD60) line was used (Hd iPSC Consortium, 2017) which was derived from an HD juvenile patient and re-programmed using integrating vectors.

HiPSC lines were cultured and differentiated as previously described (Comella-Bolla et al., 2020). In brief, cells were kept in the pluripotent state using mTeSR^TM^1 (Stem Cell Technologies, Grenoble, France) on BD Matrigel-coated plates (BD Biosciences, Oxford, Oxon, United Kingdom), and differentiated to neural progenitors using an in-house differentiation protocol as described elsewhere (Comella-Bolla et al., 2020).

### Immunocytochemistry

HiPSC-derived cultures at DIV 37 were fixed at room temperature with 4% (w/v) paraformaldehyde (Fisher Scientific UK Limited, Leicestershire, United Kingdom), washed in PBS and stored at 4°C in 0.03% Sodium-Azide (Sigma-Aldrich, Madrid, Spain) PBS until use. For immunolabeling, samples were blocked and permeabilized for 45 min with PTB solution [PBS with 0.3% Triton X-100 (Sigma-Aldrich), 0.03% Sodium-Azide, 1% BSA (Sigma-Aldrich) and/or 5% Normal Goat serum (Vector Laboratories Ltd., United Kingdom) and 5% Donkey Serum (Jackson Immuno Research Laboratories Inc.; PA, United States)], before being incubated overnight at 4°C with primary antibodies (Table 1). After overnight incubation, samples were washed with PBS. Then cells were incubated for 90 min at room temperature in darkness in smooth motion with appropriated fluorophore-conjugated secondary antibodies (Table 1). After washing in PBS, cells were counterstained with DAPI (4′,6-diamidino-2-phenylindole) for nuclear staining (Thermo Fisher Scientific, Waltham, MA, United States). Coverslips were mounted in Fluoromount-G media (Southern Biotech, AL, United States) and imaged using a Leica SP5 TCS Two-photon laser scanning confocal microscope (Leica Microsystems Heidelberg GmbH, Mannheim, Germany). FKN staining was quantified using the open-access CellProfiler software (BROAD institute, MA, United States). Three independent experiments were run in parallel for each cell line, CTR33 and HD60, from which we analyzed two replicas. Three to five pictures of fields of view were taken with the epifluorescence Leica AF600 microscope (Leica Microsystems, Wetzlar, Germany). For each picture we analyzed (1) the total area covered by MAP2B staining to assess the neuronal density and (2) the mean intensity of FKN staining.

**TABLE 1:** List of antibodies used in the study.

Antibody	Company	Dilution
**Immunocytochemistry procedure**		
Rabbit polyclonal anti-fractalkine	ABCAM, Cambridge, United Kingdom	1:250
Rabbit polyclonal anti-Iba1	Wako Chemicals, United States	1:500
Mouse anti-MAP2B (Clone 18/MAP2B)	BD Transduction Laboratories, United States	1:500
AF488 donkey Anti-Rabbit IgG (H+L)	Jackson Immuno Research, United States	1:500
Cy3 donkey Anti-Rabbit IgG (H+L)	Jackson Immuno Research, United States	1:500
AF647 Goat Anti-Mouse IgG (H+L)	Thermo Fisher Scientific, Waltham, MA, United States	1:500
**Immunohistochemistry procedure**		
Monoclonal mouse anti-PSD-95	Thermo Fisher Scientific, Waltham, MA, United States	1:1000
Polyclonal rabbit anti-Iba1	Wako Chemicals, United States	1:800
AF488 goat anti-rabbit IgG (H+L)	Jackson Immuno Research, United States	1:200
AF555 goat anti-mouse IgG (H+L)	Thermo Fisher Scientific, Waltham, MA, United States	1:500
**Western blot procedure**		
Polyclonal goat anti-fractalkine	R&D systems, United States	1:500
Polyclonal rabbit anti-β-actin	Santa Cruz Biotechnology, United States	1:20,000
HRP donkey anti-goat IgG	Promega, United States	1:2000
HRP donkey anti-rabbit IgG	Promega, United States	1:5000

### Quantitative Real-Time PCR

Aqueous phase containing total RNA was isolated from human and mouse brain regions or cells using TRI Reagent (Sigma-Aldrich) following the manufacturer’s protocol. Then total RNA was purified with Direct-zol RNA MiniPrep Plus (Zymo Research, Irvine, CA, United States). A range of 0.25 to 1 μg of RNA for each condition was reverse transcribed using a PrimeScript RT reagent kit (Takara, Japan). The RT reaction was performed at 42°C for 60 min followed by an additional 5 min at 70°C. cDNA was diluted to 5 ng/μL and 2 μL was used to perform qRT-PCR. PrimeTime qPCR assays (Table 2) were used as recommended by provider (IDT technologies, United States). qRT-PCR was carried out with Premix Ex Taq (RR390A, Takara, Japan) in 6 μL final volume using a CFX384-C1000 thermal cycler (Bio-Rad Laboratories, Madrid, Spain). The two-step amplification program was: 40 cycles of 2 min at 95°C for denaturation and polymerase activation, 5 s at 95°C for denaturation, and a final extension for 20 s at 60°C. For each target gene, the expression level was determined using a standard curve (efficiency between 93 and 100%) and normalized to housekeeper gene mRNA levels. Reactions were performed in triplicate to reduce variability. The ΔΔCt method was used to analyze the data.

**TABLE 2:** PrimeTime qPCR assays used in the study.

Gene	Assay code	RefSeq number	Species
Fractalkine	Hs.PT.56a.1173656	NM_002996	*Homo sapiens*
CX3CR1	Hs.PT.58.4589254	NM_001171174	*Homo sapiens*
GAPDH	Hs.PT.56a.40035104	NM_002046	*Homo sapiens*
Rn18s	Hs.PT.39a.22214856.g	NR_003286	*Homo sapiens*
RPL13A	Hs.PT.51.21531404	NM_012423	*Homo sapiens*
Fractalkine	Mm.PT.56a.8767901	NM_009142	*Mus musculus*
Cx3cr1	Mm.PT.56a.17555544	NM_009987	*Mus musculus*
Gapdh	Mm.PT.39a.1	NM_008084	*Mus musculus*
Rn18s	Control 18s	NR_003286	*Mus musculus*
Pcna	Mm.PT.56a.23375948	NM_011045	*Mus musculus*
C1qb	Mm.PT.58.7170292	NM_009777	*Mus musculus*
C3	Mm.PT.58.17325540	NM_009778	*Mus musculus*
Ptger4	Mn.PT.58.10399658	NM_008965	*Mus musculus*
Smad2	Mn.PT.58.12362708	NM_010754	*Mus musculus*
Smad3	Mn.PT.58.10139890	NM_016769	*Mus musculus*
Tgbr1	Mn.PT.5828402453	NM_009370	*Mus musculus*
Tyrbp	Mn.PT.58.6069426	NM_011662	*Mus musculus*

### Western Blot Analysis

Total protein extracts were obtained after organic separation and homogenization of samples in TRI Reagent (Sigma-Aldrich) following the manufacturer’s instructions. Protein concentration was determined by the Bradford assay (Bio-Rad Laboratories). Western blot analyses were performed as described previously (Comella-Bolla et al., 2019). In brief, 20 μg of total protein extracts were subjected to 10% SDS-PAGE, then proteins were transferred to Nitrocellulose membrane (Amersham Pharmacia Biotech, San Francisco, CA, United States) and probed by incubation with either anti-β-actin or anti-FKN primary antibodies, washed and incubated with appropriate secondary antibodies (Table 1). Chemiluminescent detection was performed by incubating with Luminata Classico Western HRP Substrate (Millipore, Burlington, MA, United States) and exposure to Fuji Medical X-Ray Film Super RX-N (Fujifilm, Japan). Western blot quantification was performed on digital acquired films by using ImageJ Gel Analysis. Data were expressed as the ratio between the band intensity of the protein of interest and that of β-actin. The relative ratio between FKN immunoreactive bands was also calculated.

### Immunohistochemistry and Image Analysis

Wild-type and R6/1 mice were sacrificed via cervical dislocation. Brains were isolated and post-fixed overnight with 4% paraformaldehyde, cryoprotected with 30% (w/v) sucrose in PBS, frozen in dry ice and stored at −80°C until sectioning. Serial 40 μm sections were obtained at level 2.4 mm anterior to Bregma on the cryostat, and stored in 0.02% Sodium-Azide (w/v) in PBS at 4°C until use. To label post-synaptic glutamatergic spines and microglial cells, double immunohistochemistry was performed with anti-PSD-95 and anti-Iba1 antibodies, respectively, as previously described (Espinosa-Parrilla et al., 2015). Briefly, sections were first washed in PBS containing 0.3% (v/v) Triton X-100. They were then incubated with 50 mM NH_4_Cl in PBS for 15 min. After a new washing with 0.3% Triton-X100 PBS, sections were incubated with a blocking solution of PBS containing 0.3% Triton-X100 and 10% Normal Goat Serum for 2 h. Sections were then co-incubated overnight at 4°C with both anti-Iba1 and anti-PSD-95 antibodies (Table 1). After washing in the blocking solution, sections were then incubated for 2 h with combinations of appropriate secondary antibodies (Table 1). All antibodies were diluted in the previously described blocking solution. Incubations with either mouse or rabbit IgG as primary antibodies were used for negative controls. All washes and incubations were done at room temperature, except for the primary antibody incubation, which was done at 4°C. After antibody incubations, slices were washed, incubated in DAPI, mounted with Fluoromount-G and kept in the dark.

Stereological counting of microglial cells was preformed on Iba-1 single immunostained sections. Striatal regions were outlined at 2.5× magnification on arbitrary uniform random (AUR) coronal sections. Individual cells were viewed at 40× and then counted. We applied the FIJI Auto Local Threshold plugin followed by the Analyze Particles Plugin for ImageJ to identify and analyze cells. For the microglial morphological analysis, series of confocal images from Iba-1-immunostained sections were acquired at 63× (oil immersion, 1.5 μm step size and a zoom of 1×) using a Leica SP5 Two-photon confocal microscope. Microglial morphology was semi-automatically processed in 3D using 3DMorph, a MATLAB-based program developed by York et al. (2018). For image acquisition and processing the voxel sizes were 0.241 μm in the XY plane with a 1.5 μm step size. Only the 488 nm channel (labeling the Iba-1 stained cells) was used for picture acquisition, and all cell processes (small and large) were used in our analysis. The microglial cell parameters analyzed were: cell volume, territorial volume, cell complexity (estimated as the quotient cell territory/cell volume), the number and longitude of cell processes and the number of endpoints. For every image acquired, 7–10 full cells were isolated and analyzed. To determine the relative amount of PSD-95-immunopositive puncta inside of Iba1-positive cells, confocal images were acquired at 63×, a 130 nm step size and a zoom of 3.5×. Then, images were processed with Imaris (Oxford Instruments, United Kingdom) to create a 3D reconstruction of microglia and count the post-synaptic puncta inside them (Supplementary Video). The images were prepared and rendered, and the relative amount of PSD-95 positive puncta engulfed by microglia was calculated using the engulfment assay described by Schafer et al. (2014).

### Electrophysiological Field Recordings

Wild-type and R6/1 mice were sacrified via cervical dislocation. Brains were quickly removed and immersed on oxygenated (95% O_2_, 5% CO_2_) ice-cold aCSF containing (in mM) 124 NaCl, 24 NaHCO_3_, 13 glucose, 5 HEPES, 2.5 KCl, 2.5 CaCl_2_, 1.2 NaH_2_PO_4_ and 1.3 MgSO_4_. Mouse brain sagittal sections were obtained on a vibratome (Microm HM 650 V, Thermo Fisher Scientific, Waltham, MA, United States) at 350 μm thickness in oxygenated ice-cold aCSF. Slices were then transferred to an oxygenated 32°C recovery solution of the following composition (in mM): 92 NMDG, 30 NaHCO_3_, 25 glucose, 20 HEPES, 10 MgSO_4_, 5 sodium ascorbate, 2.5 KCl, 1.2 NaH_2_PO_4_, 3 sodium pyruvate, 2 thiourea, and 0.5 CaCl_2_; at 32°C for 15 min (Choi et al., 2019). Then, slices were transferred to oxygenated aCSF at room temperature and left for at least 1 h before recording.

For recording, slices were placed in a multi electrode array (MEA) recording dish and fully submerged in oxygenated aCSF at 34°C. Electrophysiological data were recorded with a MEA set-up from Multi Channel Systems MCS GmbH (Reutlingen, Germany) composed of a 60 channels USB-MEA60-inv system with a blanking unit from Multi Channel Systems and a STG4004 current and voltage generator. Experiments were carried out with 60MEA200/30iR-ITO MEA dishes consisting of 60 planar electrodes (30 μm diameter) arranged in an 8 × 8 array (200 μm distance between neighboring electrodes). Software for stimulation, recording and signal processing were MC Stimulus and MC Rack from Multi Channel Systems. Using a digital camera during recording assessed the position of the brain slices on the electrode field and the location of the laser for stimulation.

Striatal field post-synaptic currents (fPSC) were recorded in the dorsal striatum in response to stimulation of cortical afferents. One MEA electrode located at 2.4–2.1 mm lateral to bregma, 0.8–0.6 mm anterior to bregma, and 2.2–2.3 mm depth from brain surface was set as stimulation one (Figure 5A), and fPSC were evoked by single monopolar biphasic pulses (negative/positive, 100 μs per phase). For input/output curves, corticostriatal fibers were stimulated with trains of three identical pulses at increasing currents (250–3000 μV). The pulse amplitude of subsequent stimuli was set to evoke 40% of the saturating fPSC. LTD was induced by theta-burst stimulation (TBS) consisting of 10 trains spaced 15 s apart. Each train consisted of 10 bursts at 10.5 Hz (theta), and each burst consisted of four stimuli at 50 Hz (Figure 5B). Thus, the whole TBS stimulation period lasted 2.5 min (Hawes et al., 2013). To determine the effects of microglial activity in striatal fPSC evoked by stimulation of cortical afferents, slices were incubated with either 100 μM minocycline (Mino) (Sigma-Aldrich) (Song et al., 2015; Acharjee et al., 2018) or 2 nM FKN (PeproTech, London, United Kingdom) (Bertollini et al., 2006; Liu et al., 2015; Pandur et al., 2019) from 30 min prior to and during electrophysiological recordings.

### Statistical Analysis

Data are presented as mean ± standard error of the mean (SEM). Homogeneity of variance was checked using Levene’s test. Analyses were performed by the unpaired Student’s *t*-test, analysis of variance (ANOVA), or repeated measures two-way ANOVA. Variance main components were analyzed with either genotype and age, or genotype and treatment as factors by the Bonferroni *post hoc* test. Values of *p* < 0.05 were considered significant. Analyses were performed with the SPSS Statistics v25 (IBM Corp., United States) statistical package.

## Results

### HD Induces an Early Decrease in Striatal Fractalkine Levels

**FIGURE 1:**
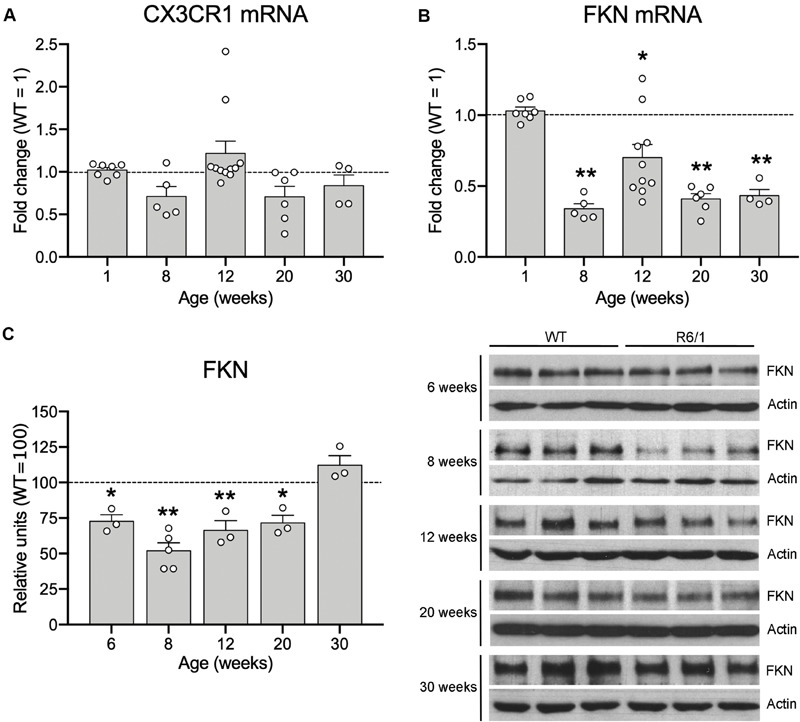
Fractalkine levels are decreased in the striatum of R6/1 mice. Histograms show the mHtt-induced fold change of CX3CR1 **(A)** and FKN **(B)** expression quantified by qRT-PCR in the striatum of R6/1 male mice relative to WT littermates (1-week WT *n* = 3, 8-week WT *n* = 5, 12-week WT *n* = 8, 20-week WT *n* = 6, 30-week WT *n* = 4; 1-week R6/1 *n* = 7, 8-week R6/1 *n* = 5, 12-week R6/1 *n* = 10, 20-week R6/1 *n* = 6, 30-week R6/1 *n* = 4) **(C)** Quantification (left) and representative immunoblots of striatal lysates (right) of FKN levels at different ages of R6/1 mice and their corresponding WT littermates. β-actin was used as loading control. Data are expressed as a percentage of WT mice. (*n* = 3 mice/group, except the 8-week R6/1 group with *n* = 5). Two-tailed unpaired *t*-test; *p < 0.05; **p < 0.01.

Since microglia have been proposed to contribute to the HD pathological process, and the FKN-CX3CR1 axis has a central role in controlling microglial activity, we first analyzed the effects of mHtt on CX3CR1 and FKN expression in the striatum of R6/1 mice at different ages (1, 8, 12, 20, and 30 weeks of age). After mRNA quantification by qRT-PCR, we found no CX3CR1 expression changes in the striatum of R6/1 mice when compared with WT animals at any of the ages studied (Figure 1A). However, we observed a significant 66% reduction in FKN expression in the striatum of R6/1 mice at 8 weeks, before disease symptoms arise, compared with WT (*t* = 18.74; *p* < 0.0001). We found a similar FKN mRNA decrease in R6/1 mice at 12, 20, and 30 weeks (*t* = 3.26, *p* = 0.010; *t* = 15.66, *p* < 0.0001; *t* = 13.45, *p* = 0.0009, respectively) (Figure 1B). When we compared FKN protein levels between WT and R6/1 mice, we observed a significant decrease in the striatal FKN concentration of R6/1 mice at 6, 8, 12, and 20 weeks (*t* = 2.599; *p* = 0.038; *t* = 5.108, *p* = 0.0002; *t* = 3.195, *p* = 0.016; and *t* = 2.700, *p* = 0.036, respectively) but not at 30 weeks of age (Figure 1C). Similarly we observed a significant 44% reduction in FKN expression in the cortex of R6/1 mice at 20 weeks (*t* = 5.299, *p* = 0.0018), whereas the CX3CR1 gene expression remained unvaried (Supplementary Figure 1).

**FIGURE 2:**
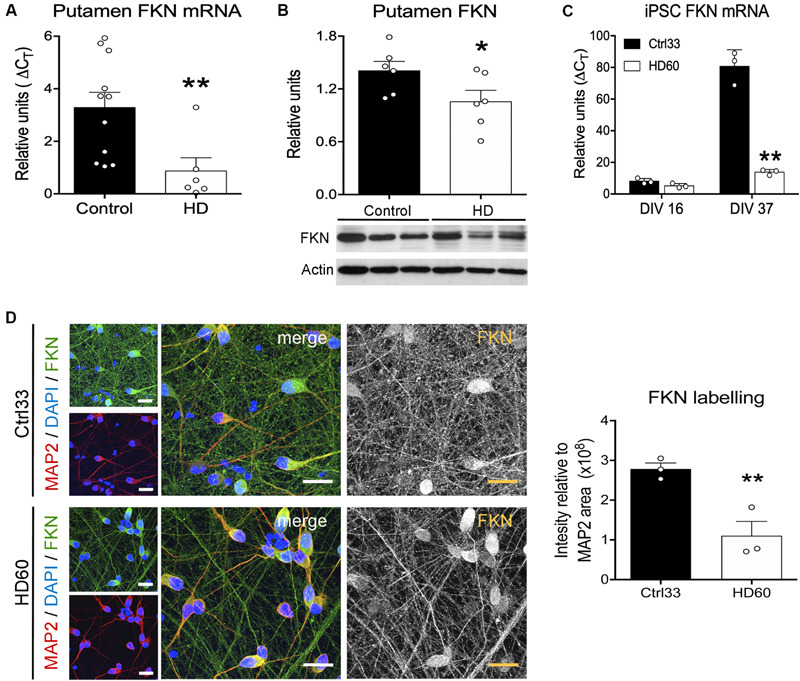
Fractalkine expression is decreased in human HD striatum and hiPSC-derived striatal neurons. **(A)** HD-induced changes of FKN expression quantified by qRT-PCR in human putamen relative to controls (HD cases *n* = 6; controls *n* = 11) **(B)** Quantification and representative immunoblots of FKN levels in human putamen. β-actin was used as loading control (*p = 0.035, Student *t*-test; *n* = 6 cases/group). **(C)** HD-induced changes of FKN expression quantified by qRT-PCR in HD60 iPSC-derived neuroblasts (DIV 16) and neurons (DIV 37) relative to Ctrl33 controls. ΔCT values are relative to housekeeping gene values (*n* = 3 cultures/group). *p < 0.05, two-tailed unpaired *t*-test; **p < 0.01, Bonferroni post-hoc test. **(D)** Confocal photomicrographs of FKN immunostaining (green) in cultured neurons (MAP2-positive; red) derived from Ctrl33 and HD60 iPSCs. Cellular nuclei are stained with Dapi (blue). Analysis of green channel picture (488 nm, black and white image, FKN) and quantification of FKN-staining intensity relative to the MAP2-stained area denotes widespread FKN staining in Ctrl33 cells, which is decreased in neurites of HD60 cells. *p < 0.05, two-tailed unpaired *t*-test (*n* = 3 cultures/condition). Scale bar: 14 μm.

We next assessed whether these FKN changes in R6/1 mice were also present in the putamen of HD patients and in striatal neurons derived from cultured HD hiPSCs. We found a significant reduction in FKN gene expression in the putamen of HD patients with respect to controls (*t* = 2.792, *p* = 0.0068) by qRT-PCR (Figure 2A). This reduction was also found when we measured the striatal protein concentration of FKN by western blot (Figure 2B). Cells derived *in vitro* from hiPSCs showed differences in FKN gene expression depending on the stage of differentiation (*F*_(1,8)_ = 165, *p* < 0.0001). We found low FKN expression in cells at neuroblast stage (DIV 16), whereas mature striatal neurons (DIV 37) presented high FKN gene expression (Figure 2C). This increase in FKN gene expression was smaller in mature neurons derived from HD hiPSCs (*F*_(1,8)_ = 122.3, *p* < 0.0001) relative to controls (Figure 2C). Immunocytochemical staining performed in cultured hiPSCs at DIV 37 showed intense FKN staining in neurites of mature neurons derived from control hiPSCs (Figure 2D). A reduction in FKN immunostaining was observed in neurites of neurons derived from the HD patient, when compared with controls (*t* = 2.188, *p* = 0.047; Figure 2D). These results indicate that asymmetric changes in the FKN-CX3CR1 axis occur during striatal HD pathogenesis, specifically modifying FKN levels.

### R6/1 Microglia Exhibit a Non-inflammatory Morphology

**FIGURE 3:**
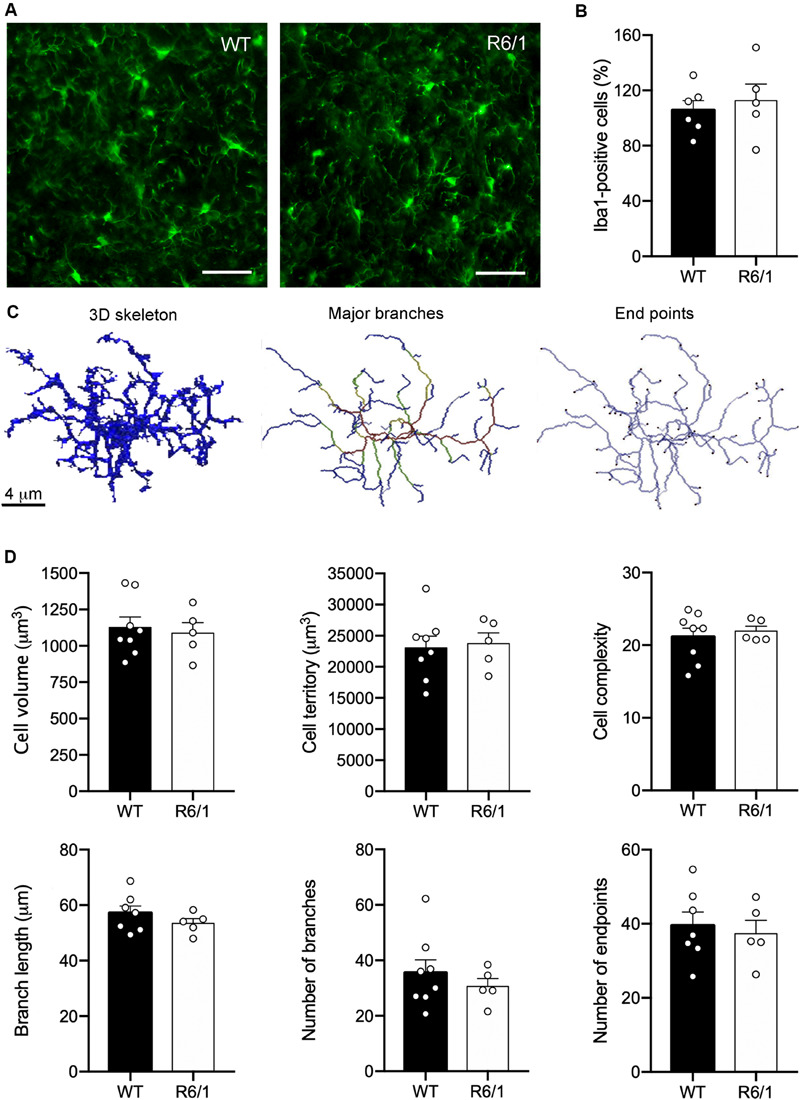
Lack of inflammatory morphology of microglia in 12-week-old R6/1 mice. **(A)** Illustrative Iba-1 immunostaining images of the striatum from WT and pre-symptomatic R6/1 mice (12 weeks of age). Scale bar: 40 μm. **(B)** Quantitative analysis of the number of Iba-1 positive cells per section. Data are expressed as a percentage of WT values (WT *n* = 3 female + 5 male mice, R6/1 *n* = 3 female + 3 male mice). **(C)** Illustrative images of 3DMorph analysis of individual microglial cells isolated from confocal microphotographs. From each full cell volume, 3D skeletons were generated and then processed to obtain an image of either major branches or branch end points. In skeleton figures, colors indicate order of connectivity (red = primary, yellow = secondary, gree*n* = tertiary, and blue = connected to end point). **(D)** Quantitative analysis of the microglial cell volume, territory occupied by each cell, microglial cell complexity (cell territory/cell volume), number and longitude of cell processes, and number of endpoints. (WT *n* = 3 female + 4 male, R6/1 *n* = 3 female + 3 male mice).

The asymmetric changes found in FKN signaling pointed to modifications in microglial activity, since microglia are the only cells in the brain parenchyma that express the FKN receptor CX3CR1 (Nishiyori et al., 1998). Therefore, FKN decrease in the striatum of R6/1 mice could potentially affect microglial behavior in this HD model. We first analyzed changes in microglial proliferation and morphology associated with classic inflammatory activation in pre-symptomatic mice (12 weeks of age) and symptomatic mice presenting motor symptoms (20 weeks of age). We found no genotype-induced changes in the number of Iba-1 immunostained microglia (Figures 3A,B). To visualize any broad morphological changes in microglia potentially indicative of a classical inflammatory response, we performed a detailed analysis with 3DMorph (Figure 3D), a sensitive program that allows analyzing many different morphological parameters (York et al., 2018). In all cases, we observed a surveillant ramified microglial shape (Figure 3C). We found no effects of phenotype or age in either the microglial cell volume, the territory occupied by the cells, the microglial cell complexity, the number and length of cell processes, or the number of endpoints (Figure 3D).

### Increased Microglial Engulfment of PSD-95-Positive Puncta in R6/1 Mice

**FIGURE 4:**
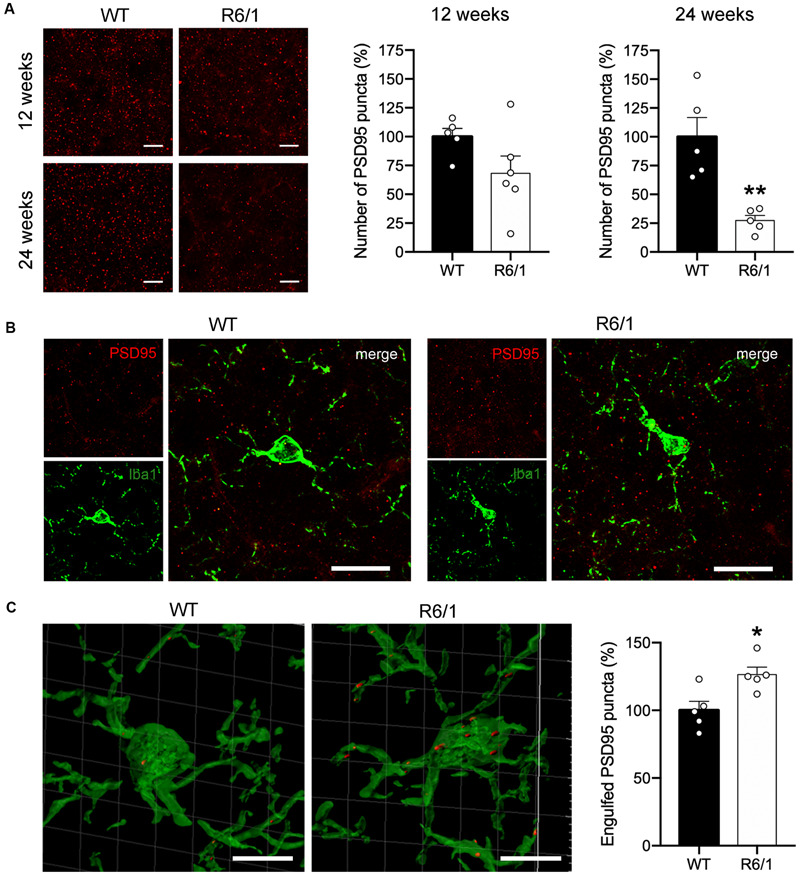
Microglia increases PSD-95-positive puncta engulfment in 12-week-old R6/1 mice. **(A)** Illustrative confocal images of PSD-95 immunostaining and quantitative analysis of the number of PSD-95-positive puncta per section in the striatum of WT and R6/1 mice of 12 and 20 weeks of age. Graph data are expressed as a percentage of WT values. **(B)** Illustrative confocal images of Iba-1 (green) and PSD-95 (red) double immunostaining in the striatum of WT and R6/1 mice at 12 weeks of age. **(C)** Three-dimensional microglia reconstruction and surface rendering by means of Imaris software showing larger relative numbers of PSD-95 positive puncta inside Iba-1 positive cells in R6/1 mice. Data are expressed as a percentage of engulfed puncta in WT striatum. *p = 0.0158; **p = 0.003, two-tailed unpaired *t*-test (*n* = 2 female + 3 male mice/group). Scale bar: 15 μm in a and b; 7 μm in c.

In spite of the absence of microglial morphological changes, FKN decrease in the striatum of R6/1 mice could potentially be affecting the non-inflammatory activity of microglia. Since FKN has been proposed to play an important role in regulating microglia-mediated synaptic pruning (Gunner et al., 2019) we estimated the density of PSD-95 immunopositive puncta in the dorsal striatum of pre-symptomatic mice (12 weeks of age) and symptomatic (20 week of age) R6/1 mice (Figure 4A). Density of PSD-95 immunopositive puncta was decreased by 71% in the dorsal striatum of R6/1 mice at 20 weeks of age (*t* = 4.205; *p* = 0.003) relative to WT, whereas the 31% reduction found at 12 weeks of age did not reach significance (*t* = 1.788; *p* = 0.107) (Figure 4A). To estimate the microglial contribution to this decrease we quantified the number of PSD-95 puncta engulfed by microglia at 12 weeks of age using a synaptic engulfment assay (Supplementary Video and Figures 4B,C). We found a 27% increase in the number of PSD-95 positive puncta inside Iba1-stained microglia in the dorsal striatum of R6/1 mice relative to WT (*t* = 3.051; *p* = 0.015). However, we found no expression changes of microglial genes involved in phagocytosis and trophic microglial activities in the striatum of 20-week-old R6/1 mice compared with WT (Supplementary Figure 2). These results point toward an abnormal increase in microglial synaptic engulfment in pre-symptomatic HD animals that could contribute to the progressive loss of synapses and striatal dysfunction.

### Microglial Activity Is Essential for Corticostriatal Synaptic Long-Term Depression

**FIGURE 5:**
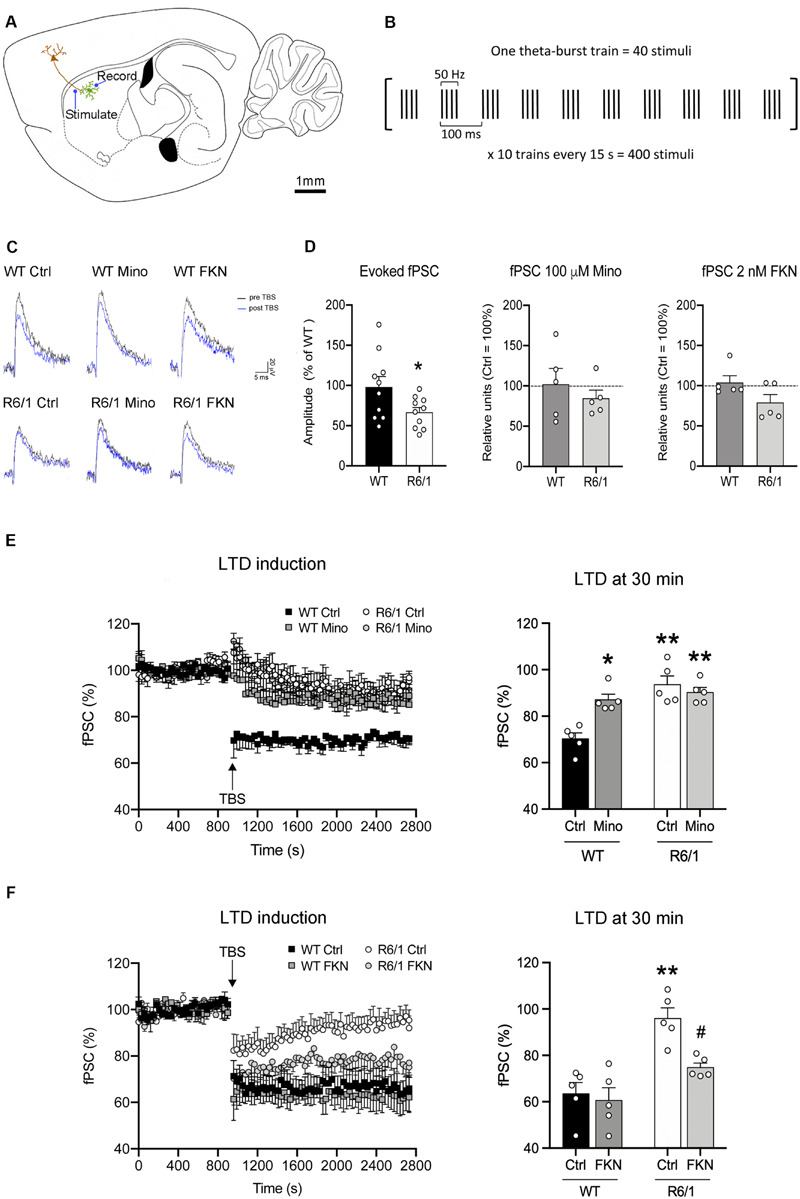
Microglia are essential for long-term depression and fractalkine restores corticostriatal synaptic plasticity in 12-week-old R6/1 mice. **(A)** Drawing of a mouse brain sagittal section showing the location of stimulating and recording electrodes for corticostriatal fPSC recordings (adapted from Paxinos Mouse Brain Atlas). **(B)** Schematic of the TBS paradigm for LTD induction. A single train of stimuli is illustrated in brackets and annotated to show the stimuli number and frequency, as well as the time scale. **(C)** Representative fPSC traces prior to TBS (pre-TBS, black) and after TBS (post-TBS, blue) in the six conditions of electrophysiological recordings. **(D)** Histograms show the quantification of fPSC amplitude in the striatum of WT and R6/1 mice relative to WT (*n* = 10 male mice/group), and in the presence of either 100 μM minocycline (Mino; *n* = 5 male mice/group) in the bath or 2 nM fractalkine (FKN; *n* = 5 male mice/group) in the bath, relative to absence in the bath (Ctrl). fPSC amplitude was calculated as the mean of 30 different fPSC evoked every 30 s during 15 min prior to TBS. One-factor ANOVA test; *p < 0.05 different from WT **(E,F)** Graphs show the time course and quantification of fPSC evoked at corticostriatal synapses and the LTD induced after a TBS (arrow) in presence or absence of either **(E)** 100 μM Mino in the bath (*n* = 5 male mice/group) or **(F)** 2 nM FKN in the bath (*n* = 5 male mice/group). fPSC amplitudes are represented as a percentage of baseline. Histograms show the mean amplitude of fPSC evoked at 29–30 min after TBS. Bonferroni’s Multiple Comparison *post hoc* test; *p < 0.05, **p < 0.01, different from WT-Ctrl; ^#^*p* < 0.05 different from R6/1-Ctrl.

We next explored whether microglial activity has a role in corticostriatal synaptic plasticity such as LTD induction, which is known to be altered in HD mice (Creus-Muncunill et al., 2019). We recorded fPSC in the dorsal striatum after electrical stimulation of cortical afferents in sagittal slices (Figure 5A). In all recordings we selected a stimulus pulse to evoke the 40% of the saturating fPSC in an input/output curve. In these conditions, the amplitude of evoked fPSC in the striatum of R6/1 mice decreased 54% compared with WT (*F*_(1,18)_ = 4.52, *p* = 0.0048), whereas the presence of either 100 μM Mino or 2 nM FKN in the bath did not modify basal fPSC amplitude (Figures 5C,D). To evaluate synaptic dysfunction, we induced LTD using a TBS protocol (Figure 5B) and compared the amplitude of evoked striatal fPSC. We analyzed the effect of microglial activity depletion over LTD by adding 100 μM Mino in the bath (Figures 5C–E). After two-way ANOVA analysis, we found fPSC amplitude differences among the four mouse groups (WT-Ctrl, WT-Mino, R6/1-Ctrl, and R6/1-Mino) 30 min after LTD induction (*F*_(3,20)_ = 13.88, *p* < 0.0001). Factor analysis showed genotype (*F*_(1,20)_ = 27.62, *p* < 0.0001) and drug (*F*_(1,20)_ = 5.838, *p* = 0.028) effects, and genotype/drug interactions (*F*_(1,20)_ = 13.174, *p* = 0.002). According to the Bonferroni post-hoc test, TBS induced a decrease in the fPSC amplitude only in WT-Ctrl mice (Figure 5E), while LTD was not induced in WT-Mino nor R6/1-Ctrl and R6/1-Mino groups. These results suggest that microglial activity is essential for corticostriatal synaptic plasticity.

### Fractalkine Restores Corticostriatal Long-Term Depression in R6/1 Mice

We then evaluated the putative role of FKN in corticostriatal LTD dysfunction in R6/1 mice by adding 2 nM FKN in the bath (Figures 5C,D,F). In these conditions, we found fPSC amplitude differences among the four mouse groups (WT-Ctrl, WT-FKN, R6/1-Ctrl, and R6/1-FKN) 30 min after LTD induction (two-way ANOVA, *F*_(3,20)_ = 9.474, *p* = 0.001). Factor analysis showed effects of genotype (*F*_(1,20)_ = 19.330, *p* = 0.0004) and treatment (*F*_(1,20)_ = 5.581, *p* = 0.031), but not genotype/treatment interaction (*F*_(1,20)_ = 3.512, *p* = 0.0792). According to the Bonferroni *post hoc* test, TBS decreased the fPSC amplitude in WT-Ctrl, WT-FKN, and in R6/1-FKN mice, although the magnitude of the induced LTD in these animals was smaller than the one observed in WT mice (Figure 5F). These results indicate that a lack of FKN is involved in striatal synaptic plasticity dysfunction in pre-symptomatic R6/1 mice.

## Discussion

Microglia have long been regarded as a mere driver of chronic inflammation in neurodegenerative diseases. With the present work, we make a meaningful contribution to the growing appreciation that their role is much more nuanced. In our HD mouse model, we found no gross, inflammatory microglial morphological changes. Instead, 3D reconstruction allowed us to visualize an increase in their engulfment of post-synaptic material, potentially driven by changes in neuronal FKN. Our data also highlight the role for microglia in altering the electrophysiological characteristics of corticostriatal synapses, specifically LTD, with FKN being central in modulating this microglial homeostatic function.

FKN-CX3CR1 signaling depletion is often correlated with increased neuroinflammation (Bachstetter et al., 2011). In response to pathogenic stimuli microglia acquire a pro-inflammatory profile and decrease CX3CR1 expression or activity (Ransohoff and Perry, 2009). Similarly, CX3CR1 polymorphisms that decrease receptor activity or affinity for FKN are linked to enhanced disease progression in amyotrophic lateral sclerosis (Lopez-Lopez et al., 2014; Calvo et al., 2018), Alzheimer’s disease (Finneran and Nash, 2019) and neuroinflammatory pathologies (Arli et al., 2013; Cardona et al., 2018). More interestingly, knocking out CX3CR1 in Alzheimer’s disease mice showed reduced Aβ deposition and increased microglial phagocytic activity (Liu et al., 2010). Also, a recent article showed that lowering CX3CR1 levels or partially inhibiting its activity in the brain might be a therapeutic strategy to increase neuronal Aβ clearance, reduce Aβ levels and delay progression of Alzheimer’s disease (Hickman et al., 2019). These studies suggest that a decrease in microglia-neuron communication, caused by a reduction of FKN-CX3CR1 signaling, is sufficient for losing the anti-inflammatory and homeostatic function of microglia, likely also affecting physiological processes (Chamera et al., 2019).

Interestingly, we found no significant changes in CX3CR1 expression in R6/1 mice over time, but instead a significant drop in FKN expression. These results are consistent with two previous gene expression analyses showing an mHtt-induced decrease in FKN expression in STHdh^Q111/Q111^ cells (Lee et al., 2007) and the striatum of 12-week-old R6/2 mice (Vashishtha et al., 2013). Additionally, in our study the FKN decrease preceded the onset of symptoms, with no inflammatory microglial morphological changes. Furthermore we observed no expression changes in any of the microglial genes analyzed in the striatum of 20-week-old, symptomatic R6/1 mice. Based on these data, pathological changes in the FKN-CX3CR1 axis could instead be driven by mHtt-induced changes in neuronal FKN production, rather than by microglial deregulation. This result is confirmed by other studies showing a lack of inflammatory markers in the striatum of non-symptomatic R6/1 mice (Pérez-Severiano et al., 2002; Renoir et al., 2015). In this line, a systems biology analysis of different HD mouse models reported wide mHtt-induced gene expression deregulation in the striatum in a CAG-length dependent manner (Veldman and Yang, 2018). In this study the main group of deregulated proteins was involved in synaptic processes and included decreased FKN expression. Furthermore, network analysis of human post-mortem expression microarrays revealed FKN as an important novel factor in HD pathogenesis and survival (Chandrasekaran and Bonchev, 2016). Further detailed studies might characterize the molecular pathways involved in the mHtt-induced FKN down-regulation.

Fractalkine has been proposed to act in the adult brain as a neuronal signal maintaining microglia in a surveillant state (Ransohoff and Perry, 2009). Conversely, other studies reported decreased release of inflammatory factors and attenuated microglial activation in the striatum of both CX3CR1-knockout (KO) and knockdown mice after experimental ischemia (Tang et al., 2014; Liu et al., 2015) and in CX3CR1-KO mixed cultures (Mattison et al., 2013). Furthermore, decreased FKN gene expression was also observed in aged BACHD mice lacking metabotropic glutamate receptor 5, which was proposed to account for decreasing microglial activation in these mice (Carvalho et al., 2019). These studies suggest instead a role for FKN in the pro-inflammatory signaling of activated microglia different to that of surveillant microglia.

Our results suggest that decreased FKN may be modifying the role that microglia play in synaptic plasticity. Moreover, it has been proposed that FKN could play an important role in regulating synapse engulfment by microglia (Gunner et al., 2019). Previous studies demonstrated a reduction of MSN spine density in the striatum of 3-month-old YAC128 mice (Marco et al., 2013) and in hippocampal apical dendrites from R6/1 mice (Miguez et al., 2015). Also in R6/1 mice, decreased PSD-95 levels, which may affect the strength and number of synaptic contacts, have been proposed to be in the basis of the functional alterations observed in HD (Nithianantharajah et al., 2008). Accordingly, PSD-95 levels are decreased in the striatum of symptomatic knock-in Hdh^Q7/Q111^ mice, and this correlates with a drop in the number of PSD-95 immunopositive puncta and altered corticostriatal long-term potentiation (Puigdellívol et al., 2015). We here describe for the first time changes in engulfment of PSD-95 immunopositive puncta by striatal microglia at pre-symptomatic stages of HD, which could contribute to the progressive loss of synapses and striatal dysfunction. Increased microglia-mediated engulfment of synaptic material has been described in mouse models of Alzheimer’s disease (Hong et al., 2016), schizophrenia (Sekar et al., 2016), and Rett syndrome (Derecki et al., 2012) via expression of traditional immune complement molecules (Hong et al., 2016; Sekar et al., 2016). These findings suggest that excessive microglia-mediated synaptic engulfment leads to synaptic dysfunction and could constitute a common pathological mechanism for many neurological and psychiatric pathologies. Whether environmental enrichment increases PSD-95 levels and improves cognition through improving microglia-neuron signaling in HD should be further explored.

Early corticostriatal LTD dysfunction in HD has been widely observed in R6/1 mice and other HD models (Cummings et al., 2007; Creus-Muncunill et al., 2019; Ghiglieri et al., 2019) and is considered one of the abnormal synaptic mechanisms of the disease (Unschuld et al., 2012; Burgold et al., 2019). We here found that depletion of microglial activity with Mino induced a decrease in corticostriatal LTD in WT mice, suggesting that microglial activity is essential for striatal synaptic function. Similar results in mouse hippocampus also suggest a role for microglia in modulating synaptic plasticity. For example, Mino has been reported to prevent the impairment of hippocampal long-term potentiation in a mouse model of Alzheimer’s disease (Wang et al., 2004) and in experimental sepsis-associated encephalopathy (Hoshino et al., 2017). Interestingly, depletion of microglial activity has also been associated with reduced synaptic pruning in the basolateral amygdala of an experimental autoimmune encephalomyelitis mouse model of multiple sclerosis (Acharjee et al., 2018). In this line, FKN-induced partial restoration of LTD in R6/1 is also a new finding, further strengthening a role for impaired neuron-microglia communication in the early striatal synaptic dysfunction characteristic of HD.

The specific mechanism linking microglial homeostatic activity with this LTD impairment is still to be determined, but a role of FKN in synaptic plasticity has been previously suggested, with controversial results. CX3CR1-deficient mice show impaired synapse development during the early post-natal period (Hoshiko et al., 2012), which is associated with weak synaptic transmission and impaired functional connectivity (Zhan et al., 2014). In this line, juvenile CX3CR1-KO mice showed increased hippocampal LTD when compared with WT, but no modifications in the adult hippocampus (Paolicelli et al., 2011). Accordingly, clinical and experimental studies suggest that mHtt-induced aberrant development is at the root of some HD functional alterations (Molero et al., 2016; Hd iPSC Consortium, 2017; Van der Plas et al., 2019), in particular cortical neurons are hyperexcitable and display dysmorphic processes (Cepeda et al., 2019). Alterations in the FKN-CX3CR1 axis could contribute to this pathological development, as this signaling system is critical for functional maturation of synapses (Hoshiko et al., 2012). Conversely, disruption of FKN-CX3CR1 signaling in adult WT mice results in hippocampal long-term potentiation impairment leading to a loss of synaptic plasticity and cognitive dysfunction (Rogers et al., 2011). This loss of hippocampal synaptic plasticity was accompanied by an increase of IL-1β expression and p38 phosphorylation (Rogers et al., 2011). Interestingly, a significant increase in phosphorylated p38 levels has also been described in the striatum of 20-week-old R6/1 mice (Saavedra et al., 2011). Thus, further studies might provide evidence on a putative mechanistic link between FKN, phosphorylated p38, IL-1β release and the loss of striatal synaptic plasticity from post-natal development and adulthood to the onset of symptoms in HD.

Another mechanism of canonical neuron-microglia communication involves the CD200-CD200R1 axis. As described for FKN, CD200 is expressed by neurons and interacts with its specific receptor CD200R1, mostly expressed by microglia in the brain (Shrivastava et al., 2012). Recent work reported increased neuronal CD200 gene expression and protein levels in the brain parenchyma, along with HD pathogenesis in R6/1 mice, but no changes in microglial CD200R1 (Comella-Bolla et al., 2019). This study suggests that neuron-microglia communication through CD200-CD200R1 interaction is not compromised, and CD200 up-regulation could represent a neurotrophic signal to sustain neuronal function in the latest stages of HD (Comella-Bolla et al., 2019). Conversely, the altered FKN-CX3CR1 axis emerges as a specific pathological mechanism in neuron-microglia communication in HD, leading to the impairment of microglial homeostatic activity.

Taken together, our results reveal a key role for FKN in the pathophysiology of HD. FKN-CX3CR1 signaling deficits in HD lead to abnormal neuron-microglia interactions, which may contribute to the early striatal synaptic plasticity dysfunction characteristic of this neurological disease. However, further functional analysis might elucidate the critical contribution of microglial phagocytic activity to the pathological synaptic loss in the striatum. Similarly, many key aspects about the role of the FKN-CX3CR1 axis in microglia-mediated synaptic pruning and plasticity during neurodegeneration remain to be clarified. Learning more about the intricate roles that microglia play outside of their classic inflammatory response to damage could give us more insight into HD pathophysiology, allowing for the exploration of new therapeutic approaches for this neurological disorder.

## Data Availability Statement

The raw data supporting the conclusions of this article will be made available by the authors, without undue reservation, to any qualified researcher.

## Ethics Statement

The studies involving human participants were reviewed and approved by the Comissió de Bioètica (CBUB) Unversitat de Barcelona, Barcelona, Spain. The patients/participants provided their written informed consent to participate in this study. The animal study was reviewed and approved by the CEEA-UB (Comitè Ètic d’Experimentació Animal de la Universitat de Barcelona) University of Barcelona, Barcelona, Spain.

## Author Contributions

AK, MS, MM, and MR contributed to the conception and design of the study. AK, MS, JA, and MR designed the experiments. AK, EG-G, MS, AC-B, and AM performed all the experiments. MS, MM, and MR performed the statistical analysis. JA, JC, and MR got financial support. AK, EG-G, and MR wrote the manuscript. All authors contributed to the manuscript revision, and read and approved the submitted version.

## Conflict of Interest

The authors declare that the research was conducted in the absence of any commercial or financial relationships that could be construed as a potential conflict of interest. The handling editor declared a shared affiliation, though no other collaboration, with the authors.

## Footnotes

1 www.clinicbiobanc.org
