# Supplementary material for: Reduced Fractalkine Levels Lead to Striatal Synaptic Plasticity Deficits in Huntington’s Disease
Source: Front Cell Neurosci. 2020 Jun 18;14:163. doi: 10.3389/fncel.2020.00163 (PMC7314984; doi:10.3389/fncel.2020.00163)
Supplement: Supplementary file 1 [file Presentation_1.pdf]

Supplementary Material

Supplementary video. Image processing procedure with Imaris to create a 3D reconstruction of microglia and count the post-synaptic puncta inside them.

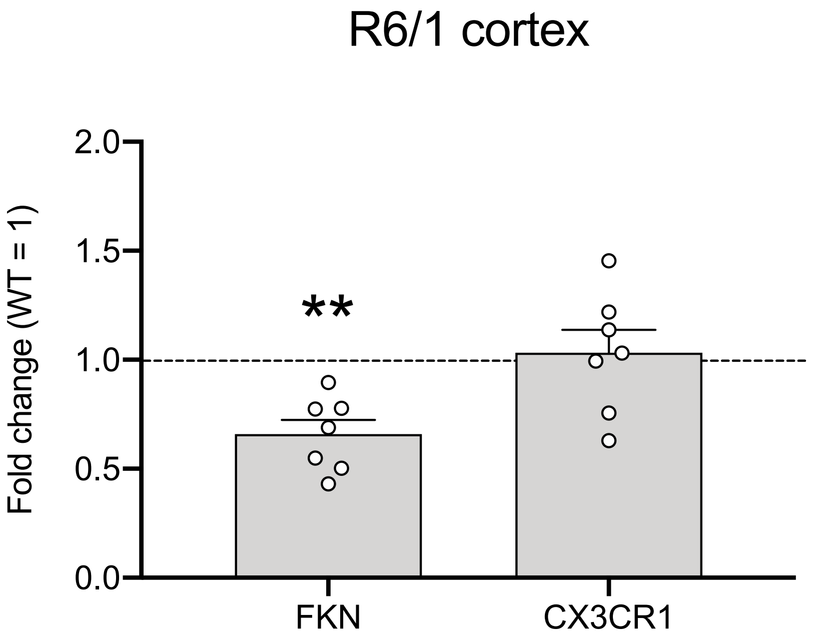

Supplementary Figure 1. qRT-PCR quantification of the expression of fractalkine and CX3CR1 genes. Histogram shows the mHtt-induced fold change of FKN and CX3CR1 gene expression quantified by qRT-PCR in the cortex of 20-week-old R6/1 male mice relative to WT littermates (n=8 WT and 7 R6/1) Two-tailed unpaired t-test; **, $p < 0.01$.

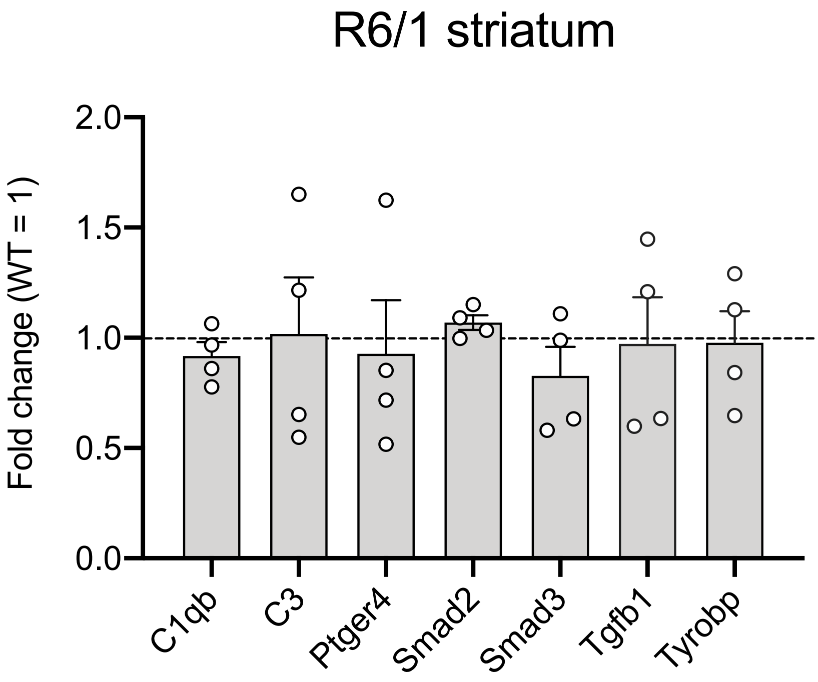

Supplementary Figure 2. qRT-PCR quantification of the expression of microglial genes involved in phagocytosis and neuroprotection. Histogram shows the the mHtt-induced changes in the expression of microglial genes involved in phagocytosis and neuroprotection in the striatum of 20-week-old R6/1 male mice relative to WT littermates (n=5 WT and 4 R6/1)
